# Genomic Prediction of Complex Phenotypes Using Genic Similarity Based Relatedness Matrix

**DOI:** 10.3389/fgene.2018.00364

**Published:** 2018-08-31

**Authors:** Ning Gao, Jinyan Teng, Shaopan Ye, Xiaolong Yuan, Shuwen Huang, Hao Zhang, Xiquan Zhang, Jiaqi Li, Zhe Zhang

**Affiliations:** National Engineering Research Center for Breeding Swine Industry, Guangdong Provincial Key Lab of Agro-Animal Genomics and Molecular Breeding, College of Animal Science, South China Agricultural University, Guangzhou, China

**Keywords:** genomic prediction, genomic selection, gene annotation, haplotype models, complex phenotypes

## Abstract

In the last years, a series of methods for genomic prediction (GP) have been established, and the advantages of GP over pedigree best linear unbiased prediction (BLUP) have been reported. However, the majority of previously proposed GP models are purely based on mathematical considerations while seldom take the abundant biological knowledge into account. Prediction ability of those models largely depends on the consistency between the statistical assumptions and the underlying genetic architectures of traits of interest. In this study, gene annotation information was incorporated into GP models by constructing haplotypes with SNPs mapped to genic regions. Haplotype allele similarity between pairs of individuals was measured through different approaches at single gene level and then converted into whole genome level, which was then treated as a special kernel and used in kernel based GP models. Results shown that the gene annotation guided methods gave higher or at least comparable predictive ability in some traits, especially in the Arabidopsis dataset and the rice breeding population. Compared to SNP models and haplotype models without gene annotation, the gene annotation based models improved the predictive ability by 0.56~26.67% in the Arabidopsis and 1.62~16.53% in the rice breeding population, respectively. However, incorporating gene annotation slightly improved the predictive ability for several traits but did not show any extra gain for the rest traits in a chicken population. In conclusion, integrating gene annotation into GP models could be beneficial for some traits, species, and populations compared to SNP models and haplotype models without gene annotation. However, more studies are yet to be conducted to implicitly investigate the characteristics of these gene annotation guided models.

## Introduction

Genomic prediction (GP) (Meuwissen et al., 2001) is a powerful tool in the fields of plant and animal breeding and human complex traits and disease risk prediction. In the past decade, a series of GP approaches have been proposed, including the maker effect methods (Meuwissen et al., 2001; Habier et al., 2011; Gianola, 2013) and genomic best linear unbiased prediction (GBLUP) (VanRaden, 2008).

Currently, standard GP models estimate marker effects and calculate individual genetic values via statistical models, but most of them pay less attention to the underlying connection between the complex genetic architecture and the often simplistic mathematical formulas. Reviewing the literatures and the biological databases, abundant of biological knowledge about trait genetic architecture, gene function, regulation patterns, and gene interaction networks have been quickly accumulated. The potential usefulness of biological knowledge to accelerate GP models has been illustrated by several studies (Zhang et al., 2014; Edwards et al., 2016; Gao et al., 2017). However, the questions about what kind of biological knowledge can be used, how to integrate the prior knowledge into GP models, and how much extra predictive ability can be obtained from the assisted information still need more investigations. Under GBLUP framework, Zhang et al. (2014) incorporated the previously reported quantitative trait loci (QTLs) collected in the animal QTLdb (http://www.animalgenome.org/QTLdb) (Hu et al., 2016) into genomic prediction model, where markers were weighted according to the frequency of corresponding genomic regions being reported likely containing QTL when constructing genomic relatedness matrix. Through this way, different variances were assumed among genomic regions and predictive ability was improved, especially for traits controlled by large effect genes (Zhang et al., 2014). Similarly, −log_10_(*p*), where *p* was the *p*-value for a marker on the outcomes of interest, was utilized into genomic prediction by weighting SNPs according to −log_10_(*p*) when constructing relatedness matrices and through which predictive ability was enhanced (de Los Campos et al., 2013; Ramstein et al., 2016). In a Bayesian model, instead of using an uniform π (the proportion of markers with zero effects) for all markers, Gao et al. (2015) transferred the GWAS *p-values* into a locus-specific π and used this genetic architecture derived π into the genomic prediction model. The predictive ability of BayesB was improved by the locus-specific π. In some of the latest publications, more types of biological knowledge were incorporated into genomic prediction by partitioning markers into classes based on their functional annotation (Morota et al., 2014; Do et al., 2015; Abdollahi-Arpanahi et al., 2016; MacLeod et al., 2016) or gene ontology categories (Edwards et al., 2016; Abdollahi-Arpanahi et al., 2017).

Compared to the pedigree BLUP (Henderson, 1975), SNP based GP models (Meuwissen et al., 2001; VanRaden, 2008) show higher predictive ability under many circumstances. In both breeding value models and marker effect models, the underlying mechanism of GP was tracing QTL effects through dense genetic markers (usually SNPs) that were in linkage disequilibrium with the potential neighbor QTLs. However, on one hand, for genes or QTLs harboring more than two alleles, the bi-allelic SNP might not be adequate for tracing the multi-allelic gene effects. On the other hand, in the breeding value GP model, the SNP derived relatedness to some extent reflect the IBS (Identity by state) rather than IBD (Identity by decent). Even though the haplotypes can neither reflect the IBD perfectly, alleles from the same haplotype are more likely to be IBD. Thus, an alternative way to the existing models is using the multi-allelic genotypes in GP by constructing haplobocks with consecutive SNPs. The benefit GP gained from haplotype models has been shown in several studies (Calus et al., 2008; Meuwissen et al., 2014; Cuyabano et al., 2015; Da, 2015; Gao et al., 2017).

In several previously proposed haplotype based GP models, “artificial markers” were constructed for each haplotype allele, and relatedness matrix was constructed by matrix product of the artificial marker matrix (Calus et al., 2008; Meuwissen et al., 2014; Cuyabano et al., 2015; Da, 2015; Gao et al., 2017), or categorical models were introduced for modeling the haplotype effects (Gao et al., 2017). Alternatively, haplotype based relatedness matrix could be built by firstly calculating a haplotype allele similarity matrix for each haploblock and then converting the allele similarity matrix into individual similarity matrix (Hickey et al., 2013). From the aspect of kernel regression (Gianola et al., 2006; Gianola and van Kaam, 2008), the similarity matrix could be treated as a specific kernel and used in GP in the framework of kernel regression.

In the haplotype models, haploblocks can be defined by considering the linkage disequilibrium among a set of consecutive SNPs (Calus et al., 2008; Cuyabano et al., 2015; Da, 2015) or the number of haplotype alleles in certain haploblock (Meuwissen et al., 2014). Recently, with the aim of defining predictors according to known functioning units, Gao et al. (2017) proposed a strategy to incorporate gene annotation into GP by restricting the haploblock to the protein coding regions. Though the predictive ability of GP models were improved by defining haplotypes according to the structural genes in many complex traits (Gao et al., 2017), more alternative approaches for building genic relatedness matrices need to be examined in order to provide more choices and gain much extra predictive ability. In this study, we (1) constructed haplotypes in the protein coding gene regions, (2) calculated genomic relatedness matrix by firstly constructing haplotype similarity matrices and then converting them into individual similarity matrices, and (3) performed GP utilizing the genic haplotype relatedness matrix. Technically, a haplotype allele similarity matrix was calculated within each haplotype block and converted into individual similarity matrix. GP was performed under the kernel regression framework by treating the individual similarity matrix as a certain kernel.

## Materials and methods

In order to build haploblocks in genic regions, SNPs were mapped to protein coding genes according to their corresponding physical positions. For each gene, haplotypes were constructed throughout the gene under consideration. Within each haplotype block, allele similarity matrix was constructed by considering the SNP matching pattern between haplotype alleles. Furthermore, the allele similarity matrix was converted into individual similarity matrix. The final relatedness matrix was calculated by averaging the similarity matrices for all haploblocks. Finally, the genic haplotype similarity based relatedness matrix was used for GP. Three populations of rice, Arabidopsis, and yellow chicken were utilized for model validation (**Table 2**). We would explain these procedures in the following sections.

### Mapping SNPs to pathways

The latest version of the gene annotation of each considered species was downloaded from Ensemble (http://www.ensembl.org) using the biomaRt package (Durinck et al., 2005, 2009) of the R statistical platform (R Development Core Team, 2016) (**Table 2**). Only genes indicated as “protein_coding” by the “gene_biotype” attribute were considered. Gene boundaries were extended by 5 kb in both upstream and downstream flanking regions to include possible regulatory elements. SNPs that were available for GP were mapped to these genic regions based on their corresponding physical positions. After the SNP mapping step, SNP sets were formed for genes with at least one mapped marker. For genes with only one mapped SNP, the corresponding haplotype block existed of only this marker. For genes with more than one mapped SNPs, phased alleles of the corresponding SNPs were combined into haplotypes with the approach described by Meuwissen et al. (2014). Briefly, haplotypes were built via the following steps.

**Initialization:** For each gene, start with the first SNP *j* = 1.
**Step 1:** Include SNP *j* + 1 into the haploblock.
**Step 2:** Determine the number of alleles of the haploblock defined by these *j* + 1 markers across the whole population.
**Step 3:** Repeat step 1 and step 2 if the number of alleles remained below a previously chosen threshold restricting the number of alleles of a haploblock (we used 10 as proposed by Meuwissen et al., 2014). Otherwise, if the number of alleles exceeded this threshold, the lastly added SNP was excluded from the current haploblock and used as the starting position of the next haploblock. Return the alleles of the current haploblock and go to the initialization step with the lastly added SNP to define the next haploblock. Repeat this procedure until all SNPs on the currently considered gene were processed.

This approach produced one or more haploblocks with at least two haplotype alleles per block for each gene. Subsequently, the genic similarity matrix could be constructed using these haplotypes.

### Genic similarity based relatedness matrices

Hickey et al. (2013) introduced three approaches for constructing haplotype allele similarity matrices. In the first strategy, similarity between pairs of haplotype alleles were measured as the proportion of matched loci in current haploblock. The second strategy took not only the proportion of matched loci, but also the length of matched segments into consideration. For more details about those two approaches, please refer to the next sections. Moreover, the allele frequencies were further considered in a third strategy. The former two strategies were used in this study to construct allele similarity matrices and further convert into individual similarity matrices. The third approach was not used in the present study. Because its performance was not better than others, and it needs to use the allele frequency, which could not be estimated accurately from small populations. In the following, we illustrated the procedures for calculating allele similarity and individual similarity matrices with a small example. Table 1 showed the genotypes of five individuals and 10 consecutive SNPs from a certain gene. The SNP genotypes were phased and four different haplotype alleles were defined by these markers.

**Table 1:** Genotype matrix of five individuals and 10 consecutive markers from a certain protein coding gene.

**Individuals**	**Gamete**	**Haplotypes**	**SNPs mapped to gene**									
			***M1***	***M2***	***M3***	***M4***	***M5***	***M6***	***M7***	***M8***	***M9***	***M10***
*id1*	Paternal	*hap 1*	1	0	1	1	1	1	0	1	0	0
	Maternal	*hap 4*	0	1	1	1	0	0	1	0	1	0
*id2*	Paternal	*hap 2*	0	0	0	1	0	0	1	0	1	0
	Maternal	*hap 1*	1	0	1	1	1	1	0	1	0	0
*id3*	Paternal	*hap 3*	1	1	1	0	0	0	1	1	0	1
	Maternal	*hap 4*	0	1	1	1	0	0	1	0	1	0
*id4*	Paternal	*hap 2*	0	0	0	1	0	0	1	0	1	0
	Maternal	*hap 2*	0	0	0	1	0	0	1	0	1	0
*id5*	Paternal	*hap 2*	0	0	0	1	0	0	1	0	1	0
	Maternal	*hap 3*	1	1	1	0	0	0	1	1	0	1

*A haplotype block contains four haplotype alleles is defined by these 10 consecutive markers from a protein coding gene*.

The first strategy calculated haplotype similarity by counting the number of matched SNPs between haplotypes and dividing by the total number of markers contained in the haplotype. In a formula form, the haplotype similarity score was calculated as $h_{1}=\frac{n_{s}}{N}$, where *h*_1_ was the similarity score, *n*_*s*_ was the number of matched SNPs between two haplotypes, and *N* was the number of SNPs in current haplotype block. For example, *hap1* and *hap2* in Table 1 shared the same SNP alleles for markers *M2, M4*, and *M10*. The similarity between *hap1* and *hap2* was calculated as 3/10 = 0.3. The similarity score between a haplotype and itself equaled to 1. Therefore, similarity matrix of the four haplotypes shown in Table 1 was calculated as **H**_1_.

$$\begin{array}{l} {\text{H}}_{1}=\left[\begin{array}{cc} \begin{array}{cc} 1 & 0.3\\ 0.3 & 1 \end{array} & \begin{array}{cc} 0.4 & 0.3\\ 0.3 & 0.8 \end{array}\\ \begin{array}{cc} 0.4 & 0.3\\ 0.3 & 0.8 \end{array} & \begin{array}{cc} 1 & 0.5\\ 0.5 & 1 \end{array} \end{array}\right] \end{array}$$

In the second strategy, the measurement of haplotype similarity took the length of matching segments into account, where the similarity score increased as the number of consecutive matching SNPs increased. For certain pairs of haplotypes, the final similarity was the sum of all matched segments. Within each segment, the similarity score was calculated as the squared numbers of matching SNPs. Segments containing only one matching SNP was scored one. The overall similarity scores were further standardized by dividing the scores by the maximum of the similarity scores and taking the square root to ensure values with the scale of [0,1]. In a formula form, the haplotype similarity score was calculated as $h_{2}=\sqrt{\frac{\overset{L}{\underset{l=1}{\sum }}n_{sl}^{2}}{N^{2}}}$, where *L* was the number of matched segments between pairs of haplotypes, *n*_*sl*_ was the number of matched SNPs in the *lth* segment, and *N* was the number of SNPs in current haplotype block. For example, *hap2* and *hap4* in Table 1 shared two matching segments, the first segment contained one marker (M1) and the second segment contained seven markers (M4~M10). The similarity scores of the two segments were 1^2^ = 1 and 7^2^ = 49, respectively. Therefore, the final similarity between *hap2* and *hap4* was $\sqrt{\left(1+49\right)/100}=0.71$. The similarity matrix of the four haplotypes in Table 1 was represented in **H**_2_.

$$\begin{array}{l} {\text{H}}_{2}=\left[\begin{array}{cc} \begin{array}{cc} 1 & 0.17\\ 0.17 & 1 \end{array} & \begin{array}{cc} 0.24 & 0.22\\ 0.30 & 0.71 \end{array}\\ \begin{array}{cc} 0.24 & 0.30\\ 0.22 & 0.71 \end{array} & \begin{array}{cc} 1 & 0.36\\ 0.36 & 1 \end{array} \end{array}\right] \end{array}$$

For comparison, a third similarity matrix, where diagonal of the similar ity matrix were 1 (the similarity between two exactly same haplotypes) but the off-diagonals were zeros, was constructed. Similarity matrix for the four haplotypes in Table 1 was shown in H_*I*_.

$$\begin{array}{l} {\text{H}}_{\text{I}}=\left[\begin{array}{cc} \begin{array}{cc} 1 & 0\\ 0 & 1 \end{array} & \begin{array}{cc} 0 & 0\\ 0 & 0 \end{array}\\ \begin{array}{cc} 0 & 0\\ 0 & 0 \end{array} & \begin{array}{cc} 1 & 0\\ 0 & 1 \end{array} \end{array}\right] \end{array}$$

The next step was transferring the haplotype similarity matrix into individual relatedness matrix. For each pair of individuals, the similarity scores of the four haplotypes harbored by the two individuals were extracted from the haplotype similarity matrices (one of H_*I*_, **H**_1_, and **H**_2_) and the relatedness between the two individuals was calculated by summing up the pair-wise haplotype alleles similarity scores among the four haplotype alleles and divided by two. Let $W=\left[\begin{array}{cc} h_{id1_{P}id2_{P}} & h_{id1_{P}id2_{M}}\\ h_{id1_{M}id2_{P}} & h_{id1_{M}id2_{M}} \end{array}\right]$ denoted the similarity matrix of the four haplotypes carried by a pair of individuals (*id*_1_ and *id*_2_), where subindexes P and M denoted paternal and maternal haplotype alleles, respectively. The similarity score between *id*_1_ and *id*_2_ was calculated as $S=\frac{h_{{id1}_{P}{id2}_{P}}+\;h_{{id1}_{P}{id2}_{M}}+h_{{id1}_{M}{id2}_{P}}+h_{{id1}_{M}{id2}_{M}}}{2}$. For example, *id1* in Table 1 carried *hap1* and *hap4* while *id2* carried *hap2* and *hap1*. The similarity scores of these four haplotypes according to **H**_**2**_ were $\text{W}=\left[\begin{array}{rr} 0.17 & 1\\ 0.71 & 0.22 \end{array}\right]$ thus the relatedness between *id1* and *id2* was calculated as $G_{HAP_{id1.id2}}=\frac{\left(0.17+1+0.71+0.22\right)}{2}=1.05$. Subsequently, according to **H**_2_, the relatedness matrix of individuals shown in Table 1 could be constructed as **G**_*HAP*_. Relatedness matrices based on other types of haplotype similarity matrices could be calculated in a similar way.

$$\begin{array}{l} {\text{G}}_{HAP}=\left[\begin{array}{cc} \begin{array}{cc} 1.22 & 1.05\\ 1.05 & 1.17 \end{array} & \begin{array}{ccc} 0.91 & 0.88 & 0.74\\ 0.74 & 1.17 & 0.86 \end{array}\\ \begin{array}{cc} 0.91 & 0.74\\ 0.88 & 1.17\\ 0.74 & 0.86 \end{array} & \begin{array}{ccc} 1.36 & 1.01 & 1.18\\ 1.01 & 2.00 & 1.30\\ 1.18 & 1.30 & 1.30 \end{array} \end{array}\right] \end{array}$$

The procedures described above constructed the relatedness matrix for one genic haploblock. In practice, relatedness matrices based on the other haploblocks could be built through these procedures and the final genic relatedness matrix was obtained by averaging over the haploblock relatedness matrices. For variance components estimation and genomic prediction, the final relatedness matrix could be easily standardized by dividing the matrix by the maximum of the elements.

### Genomic prediction models

The statistical model for GP used in this study was

(1) $$\begin{array}{r} \text{y}=1_{n}\mu +\text{Z}\text{g}+\text{e}, \end{array}$$

where **y** was a vector of the observations; **1**_*n*_ was a *n*×1 vector with all elements equal to one; μ was the overall mean; **Z** was the design matrix allocates observations to genetic values; $\text{g}\sim N\left(0,\text{K}\sigma_{g}^{2}\right)$ was the genetic values; **K** was the relatedness matrices; $\sigma_{g}^{2}$ was the variance of genetic values; $\text{e}\sim N\left(0,\text{I}\sigma_{e}^{2}\right)$ was the residuals; **I** was the identity matrix and $\sigma_{e}^{2}$ was the residua variance.

We compared the newly proposed approaches to the standard GBLUP (VanRaden, 2008). In GBLUP, the genomic relatedness matrix was calculated as $\text{G}=\frac{\text{M}{\text{M}}^{\prime }}{2\overset{m}{\underset{k=1}{\sum }}p_{k}\left(1-p_{k}\right)}$, where **M** was the minor allele frequency (MAF) adjusted genotype matrix with elements (0−2*p*_*j*_), (1−2*p*_*j*_), and (2−2*p*_*j*_) representing genotypes AA, AB, and BB, respectively; *p*_*j*_ was the MAF of the *jth* SNP.

For the genic similarity based models, relatedness matrices were constructed through the procedures described above. These three genic similarity based haplotype models for genomic prediction given gene annotation were denoted as *G*_*HAPI*_|*GA*, *G*_*HAP*1_|*GA*, and *G*_*HAP*2_|*GA*. For comparison, haplotype similarity based relatedness matrices without gene annotation were also calculated. Different from the gene annotation guided approaches, the naïve haplotype models constructed haploblocks for each chromosome starting from the first SNP and the rest steps were the same as genic haplotypes (Meuwissen et al., 2014). The corresponding models without gene annotation were denoted as *G*_*HAPI*_, *G*_*HAP*1_, and *G*_*HAP*2_, respectively.

For all models, variance components were estimated with the *regress* package (Clifford and McCullagh, 2014) in the R platform and genetic values were obtained by solving the mixed model equations.

### Assess of genomic predictive ability

Performance of all models were assessed through a 20 times of five-fold cross validation. Variance components were estimated in the training population and genetic values of the test population were predicted via the fitted models. Predictive ability was calculated as the Pearson's correlation between the predicted genetic values and the phenotypic values that pre-adjusted for fixed effects.

### Datasets

#### Rice

Genotypes and phenotypes of the rice breeding population were available from the rice diversity panel (https://ricediversity.org) (Begum et al., 2015; Spindel et al., 2015). Briefly, 315 elite rice breeding lines from the International Rice Research Institute (IRRI) irrigated rice breeding program was presented in this rice dataset. Several important traits such as plant height (PH), flower time (FLW), and grain yield (YLD) were tested and recorded in years 2009–2012, including wet and dry seasons each year. Totally, 58,227 SNPs passed the quality control step and were remained for further analysis. The annotations of the latest version of rice genome (*Oryza sativa Japonica Group, Build 4.0*) were downloaded from *Ensemble* via *biomaRt* (Durinck et al., 2005, 2009) R package (Table 2).

**Table 2:** Datasets description.

**Datasets**	**^#^ of observations**	**^#^ of markers**	**Reference genome**	**^#^ of mapped SNPs**	**^#^ of represented genes**	**^#^ of haplotypes**
Rice	315	58,227	*Oryza sativa* Japonica Group (Build 4.0)	44,831	22,509	25,453
Arabidopsis	349	208,481	*Arabidopsis thaliana* (assembly TAIR10.1)	193,646	27,169	167,837
Chicken	435	408,715	*Gallus gallus* (assembly GGA 5)	233,417	17,686	45,470

# Denoted “the number.”

#### Arabidopsis

The Arabidopsis population consisted of 349 natural accessions collected worldwide (Li et al., 2010; Horton et al., 2012; Kooke et al., 2016). Seeds of all accessions were genotyped with 215 K single nucleotide polymorphisms (SNPs; Li et al., 2010; Horton et al., 2012). Three replicates of each accession were cultured and transplanted under the same environmental conditions (Kooke et al., 2016). Lots of developmental traits were measured on all individual plants. Traits used for model comparisons in this study include: leaf area before vernalization (LAbv), leaf area after vernalization (LAav), flowering time (FT), petiole to leaf length ratio (PL/LL), petiole length (PL), leaf length (LL), rosette branching (RB), main stem branching (MSB), plant height at 1st silique (PH1S), total plant height (TPH), relative growth rate before vernalization (RGRbv), and relative growth rate after vernalization (RGRav).

#### Yellow chicken

The yellow chicken population used in this study was derived from a Chinese indigenous breed and maintained by Wens Nanfang Poultry Breeding Co. Ltd. (Xinxing, P.R. China) (Zhang et al., 2017; Ye et al., 2018). The population consisted of 435 males, which were the 3rd batch of the 25th generation of the population. These birds came from a mixture of full sib and half sib families with the mating of 30 males and 360 females from the 24th generation. After hatching, all birds were maintained in a closed building under controlled environmental conditions and provided with a standard diet till the end of 4 weeks of age. These birds were randomly allocated to three pens for growth performance test from 5 to 13 weeks of age, providing food and water *ad libitum*. After the growth test, all birds were slaughtered at the age of 91 days. Seventeen traits including average daily gain (ADG), average daily feed intake (ADFI), residual feed intake (RFI), and intestine length (IL) were used for model validation in this study. All individuals were genotyped with the commercially available 600 K Affymetrix Axion HD genotyping array using DNA extracted from blood samples. The phenotypes were pre-adjusted for the fixed pen effect via the flowing statistical model:

$$\begin{array}{l} \text{y}=\text{X}\text{b}+\text{Z}\text{u}+\text{e}, \end{array}$$

where **y** was a vector the raw phenotypes; **X** and **Z** were design matrices; **b** was a vector of the fixed pen effects; $\text{u}\sim N\left(0,\text{G}\sigma_{u}^{2}\right)$ was the vector of genetic values; **G** was the SNP derived relatedness matrix (VanRaden, 2008); $\sigma_{u}^{2}$ was the additive genetic variance; $\text{e}\sim N\left(0,\;I\sigma_{e}^{2}\right)$ was the vector of residuals; $\sigma_{e}^{2}$ was the residual variance and **I** was the identity matrix. The adjusted phenotypes $\bar{\text{Y}}=\text{y}-\text{X}\hat{\text{b}}$ were used as model response in the genomic prediction models.

## Results

### Predictive ability in the rice population

Predictive ability of all models in the rice breeding population was shown in Table 3 and Figure S1. Overall, the gene annotation based haplotype models (~|GA models) outperformed *GBLUP* and the naïve haplotype models to some extent. Among the three gene annotation based haplotype models, *G*_*HAPI*_|*GA*, where an identity matrix was used to measure similarity between pairs of haplotype alleles, performed best in respect of predictive ability. For plant height, *G*_*HAPI*_|*GA* showed the highest predictive ability. Compared to *GBLUP*, 4.73 and 6.43% extra accuracy were obtained by incorporating gene annotation in a haplotype model for dry season (DS_PH) and wet season (WS_PH), respectively; *G*_*HAPI*_|*GA* improved 2.21% (DS_PH) and 3.43% (WS_PH) of the predictive ability compared to the naïve haplotype model *G*_*HAPI*_. For flowering time, *G*_*HAPI*_|*GA* was 5.62 and 7.07% higher than *GBLUP* in respect of predictive ability in dry season and wet season, respectively; *G*_*HAPI*_|*GA* outperformed *G*_*HAPI*_ by 1.62 and 2.67% in DS_FLW and WS_FLW, respectively. For grain yield, *G*_*HAPI*_|*GA* showed the highest predictive ability in DS_YLD, which was 8.30 and 9.82% higher than *GBLUP* and *G*_*HAPI*_, respectively; *G*_*HAP*1_|*GA* showed the best predictive ability in WS_YLD, which was 9.30% and 16.53% higher than *GBLUP* and *G*_*HAPI*_, respectively.

**Table 3:** Pearson's correlation between observed and predicted phenotypes in the rice breeding population (Mean ± SE).

**Traits**	**GBLUP^a^**	***G*_*HAPI*_^b^**	***G*_*HAPI*_|*GA*^c^**	***G*_*HAP*1_^b^**	***G*_*HAP*1_|*GA*^c^**	***G*_*HAP*2_^b^**	***G*_*HAP*2_|*GA*^c^**
DS_PH	0.486 ± 0.007	0.498 ± 0.007	**0.509** ±**0.007**	0.493 ± 0.007	0.501 ± 0.007	0.498 ± 0.007	0.503 ± 0.007
DS_FLW	0.534 ± 0.005	0.555 ± 0.005	**0.564** ±**0.005**	0.530 ± 0.005	0.552 ± 0.005	0.540 ± 0.005	0.553 ± 0.005
DS_YLD	0.289 ± 0.006	0.285 ± 0.006	**0.313** ±**0.006**	0.286 ± 0.006	0.312 ± 0.006	0.286 ± 0.006	0.311 ± 0.006
WS_PH	0.482 ± 0.006	0.496 ± 0.005	**0.513** ±**0.005**	0.489 ± 0.006	0.507 ± 0.005	0.492 ± 0.006	0.509 ± 0.005
WS_FLW	0.467 ± 0.007	0.487 ± 0.006	**0.500** ±**0.006**	0.465 ± 0.006	0.491 ± 0.006	0.474 ± 0.006	0.492 ± 0.006
WS_YLD	0.258 ± 0.007	0.242 ± 0.007	0.268 ± 0.008	0.264 ± 0.007	**0.282** ±**0.008**	0.256 ± 0.007	0.280 ± 0.008

*For each trait (row), the values in boldface indicate the best prediction among all models. DS, dry season; WS, wet season; PH, plant height; FLW, flower time; YLD, grain yield*.

a *Genomic best linear unbiased prediction (VanRaden, 2008)*.

b *Haplotype similarity based models without gene annotation. HAPI, HAP1, and Hap2 are differ on the way of evaluating haplotype similarity*.

c *~|GA denoted gene annotation guided GP models*.

### Predictive ability in the arabidopsis population

Table 4 and Figure S2 showed the predictive ability in the Arabidopsis population. Overall, gene annotation based haplotype models outperformed *GBLUP* and the naïve haplotype models in 8 out of 12 traits. For Laav, PH1S, MSB, FT, and RGRbv, *G*_*HAPI*_|*GA* showed the best performance in respect of predictive ability and outperformed *GBLUP* by 5.47, 13.61, 5.59, 3.58, and 26.67%, respectively. For LL, PL, and RGRav, *G*_*HAP*1_|*GA* showed the best performance and outperformed *GBLUP* by 0.56%, 1.98% and 9.78%, respectively. However, *G*_*HAPI*_, *G*_*HAP*1_, and *G*_*HAP*2_, in which gene annotation information was not integrated, outperformed *GBLUP* and the gene annotation based haplotype models (~|GA) for the traits RB, PL/LL, and TPH.

**Table 4:** Pearson's correlation between observed and predicted phenotypes in the Arabidopsis population (Mean ± SE).

**Traits**	**GBLUP^a^**	***G*_*HAPI*_^b^**	***G*_*HAPI*_|*GA*^c^**	***G*_*HAP*1_^b^**	***G*_*HAP*1_|*GA*^c^**	***G*_*HAP*2_^b^**	***G*_*HAP*2_|*GA*^c^**
Labv	0.163 ± 0.009	0.161 ± 0.009	0.170 ± 0.009	0.164 ± 0.009	**0.176** ±**0.009**	0.166 ± 0.009	0.174 ± 0.009
Laav	0.201 ± 0.006	0.205 ± 0.006	**0.212** ±**0.005**	0.200 ± 0.006	0.209 ± 0.006	0.201 ± 0.006	0.208 ± 0.006
PH1S	0.191 ± 0.005	0.196 ± 0.005	**0.217** ±**0.005**	0.190 ± 0.005	0.213 ± 0.005	0.191 ± 0.005	0.211 ± 0.005
TPH	0.185 ± 0.007	0.183 ± 0.007	0.175 ± 0.007	**0.186** ±**0.007**	0.181 ± 0.007	0.185 ± 0.007	0.179 ± 0.007
MSB	0.340 ± 0.004	0.346 ± 0.004	**0.359** ±**0.004**	0.337 ± 0.004	0.346 ± 0.004	0.337 ± 0.004	0.348 ± 0.004
RB	0.281 ± 0.006	**0.289** ±**0.007**	0.283 ± 0.007	0.281 ± 0.007	0.277 ± 0.006	0.282 ± 0.006	0.276 ± 0.006
LL	0.356 ± 0.006	0.355 ± 0.005	0.353 ± 0.005	0.356 ± 0.006	**0.358** ±**0.006**	**0.358** ±**0.006**	0.357 ± 0.005
PL	0.303 ± 0.006	0.301 ± 0.006	0.301 ± 0.005	0.305 ± 0.006	**0.309** ±**0.006**	0.306 ± 0.006	0.307 ± 0.006
PL/LL	0.255 ± 0.009	0.249 ± 0.009	0.237 ± 0.008	**0.258** ±**0.010**	0.247 ± 0.008	0.257 ± 0.009	0.245 ± 0.008
FT	0.643 ± 0.003	0.653 ± 0.003	**0.666** ±**0.003**	0.642 ± 0.003	0.658 ± 0.003	0.644 ± 0.003	0.660 ± 0.003
RGRbv	0.045 ± 0.007	0.050 ± 0.007	**0.057** ±**0.007**	0.042 ± 0.007	0.054 ± 0.008	0.042 ± 0.007	0.054 ± 0.008
RGRav	0.184 ± 0.006	0.179 ± 0.006	0.194 ± 0.006	0.184 ± 0.006	**0.202** ±**0.006**	0.183 ± 0.006	0.199 ± 0.006

*For each trait (row), the values in boldface indicate the best prediction among all models. LAbv, leaf area before vernalization; LAav, leaf area after vernalization; FT, flowering time; PL/LL, petiole to leaf length ratio; PL, petiole length; LL, leaf length; RB, rosette branching; MSB, main stem branching; PH1S, plant height at 1st silique; TPH, total plant height; RGRbv, relative growth rate before vernalization; RGRav, relative growth rate after vernalization*.

a *Genomic best linear unbiased prediction (VanRaden, 2008)*.

b *Haplotype similarity based models without gene annotation. HAPI, HAP1, and Hap2 are differ on the way of evaluating haplotype similarity*.

c *~|GA denoted gene annotation guided GP models*.

### Predictive ability in the yellow chicken population

Table 5 and Figure S3 showed the predictive ability in the yellow chicken population. The haplotype models benefit from gene annotation information in six (MTW, MTMW, RFI, EW, DW, and BW45) out of 17 traits, where gene annotation models outperformed *GBLUP* and naïve haplotype models by 0.40~3.43%. For ADG, ADFI, EWG, BMW, AFW, and IL, *GBLUP* showed the best performance, while haplotype models with or without gene annotation did not show any extra gain in respect of predictive ability. For RFI and FCR, *G*_*HAPI*_ was slightly better than *GBLUP*.

**Table 5:** Pearson's correlation between observed and predicted phenotypes in the yellow chicken population (Mean ± SE).

**Traits**	**GBLUP^a^**	***G*_*HAPI*_^b^**	***G*_*HAPI*_|*GA*^c^**	***G*_*HAP*1_^b^**	***G*_*HAP*1_|*GA*^c^**	***G*_*HAP*2_^b^**	***G*_*HAP*2_|*GA*^c^**
ADG	**0.351** ±**0.005**	0.344 ± 0.005	0.342 ± 0.004	0.345 ± 0.005	0.345 ± 0.004	0.345 ± 0.005	0.345 ± 0.004
ADFI	**0.440** ±**0.004**	0.437 ± 0.004	0.438 ± 0.004	0.436 ± 0.004	0.439 ± 0.004	0.437 ± 0.004	**0.440** ±**0.004**
MTW	0.322 ± 0.005	0.315 ± 0.004	**0.328** ±**0.004**	0.314 ± 0.005	0.325 ± 0.004	0.316 ± 0.005	0.326 ± 0.004
MTMW	0.322 ± 0.005	0.315 ± 0.004	**0.328** ±**0.004**	0.314 ± 0.005	0.325 ± 0.004	0.316 ± 0.005	0.327 ± 0.004
RFI	0.464 ± 0.005	**0.468** ±**0.005**	**0.468** ±**0.005**	0.465 ± 0.005	0.466 ± 0.005	0.467 ± 0.005	0.467 ± 0.005
FCR	0.288 ± 0.004	**0.289** ±**0.004**	0.274 ± 0.004	0.286 ± 0.004	0.271 ± 0.004	0.288 ± 0.004	0.273 ± 0.004
EWG	**0.257** ±**0.009**	0.253 ± 0.009	0.256 ± 0.009	0.253 ± 0.009	0.256 ± 0.008	0.254 ± 0.009	0.256 ± 0.009
EW	0.253 ± 0.009	0.249 ± 0.010	0.253 ± 0.009	0.250 ± 0.010	**0.254** ±**0.009**	0.250 ± 0.010	**0.254** ±**0.009**
BMW	**0.144** ±**0.011**	0.142 ± 0.011	0.138 ± 0.011	0.144 ± 0.011	0.142 ± 0.011	0.143 ± 0.011	0.141 ± 0.011
BMP	0.128 ± 0.011	0.128 ± 0.011	0.123 ± 0.011	**0.130** ±**0.011**	0.128 ± 0.011	0.129 ± 0.011	0.126 ± 0.011
DW	0.175 ± 0.010	0.172 ± 0.010	0.176 ± 0.010	0.175 ± 0.010	**0.181** ±**0.009**	0.174 ± 0.010	0.179 ± 0.010
DP	0.128 ± 0.011	0.128 ± 0.011	0.123 ± 0.011	**0.130** ±**0.011**	0.128 ± 0.011	0.129 ± 0.011	0.126 ± 0.011
AFW	**0.114** ±**0.009**	0.108 ± 0.009	0.104 ± 0.009	0.112 ± 0.009	0.110 ± 0.009	0.111 ± 0.009	0.108 ± 0.009
AFP	0.128 ± 0.011	0.128 ± 0.011	0.123 ± 0.011	**0.130** ±**0.011**	0.128 ± 0.011	0.129 ± 0.011	0.126 ± 0.011
GW	0.067 ± 0.011	0.070 ± 0.010	0.066 ± 0.011	**0.071** ±**0.010**	0.068 ± 0.011	0.070 ± 0.011	0.067 ± 0.011
IL	**0.045** ±**0.005**	0.041 ± 0.005	0.037 ± 0.005	0.043 ± 0.005	0.040 ± 0.005	0.043 ± 0.005	0.039 ± 0.005
BW45	0.307 ± 0.005	0.306 ± 0.005	**0.309** ±**0.005**	0.303 ± 0.005	0.302 ± 0.005	0.304 ± 0.005	0.304 ± 0.005

*For each trait (row), the values in boldface indicate the best prediction among all models. ADG, Average daily gain; ADFI, Average daily feed intake; MTW, Mid-term body weight; MTMW, Mid-term metabolic body weight; RFI, Residual feed intake; FCR, Feed conversion rate; EWG, Eviscerated weight with giblet; EW, Eviscerated weight; BMW, Breast muscle weight; BMP, Breast muscle percentage; DW, Drumstick weight; DP, Drumstick percentage; AFW, Abdominal fat weight; AFP, Abdominal fat percentage; GW, Gizzard weight; IL, intestine length; BW45, body weight at 45 day*.

a *Genomic best linear unbiased prediction (VanRaden, 2008)*.

b *Haplotype similarity based models without gene annotation. HAPI, HAP1, and Hap2 are differ on the way of evaluating haplotype similarity*.

c *~|GA denoted gene annotation guided GP models*.

## Discussion

In this study, SNPs were mapped to protein coding genes according to the physical positions and used for haplotype construction. Different from our previous study (Gao et al., 2017), in which genic region haplotypes were encoded in both a numerical and a categorical strategy, here we constructed individual similarity matrices from the haplotype allele similarity matrices via strategies described by Hickey et al. (2013). Three strategies were utilized to calculate similarity scores between haplotype alleles. Individual similarity matrices were constructed by averaging the haplotype similarity among all genes or genome regions and used in the genetic evaluations.

Generally, the gene annotation based haplotype models proposed in this study potentially improved the genomic predictive ability. In the three datasets of rice, Arabidopsis, and yellow chicken, gene annotation based models improved the predictive ability in several traits, especially traits in the rice breeding population (Table 3 and Figure S1) and the Arabidopsis population (Table 4 and Figure S2), compared to GBLUP model. Results in the rice dataset showed that incorporating gene annotation in a haplotype model could improve the predictive ability. However, the extent of improvement was slightly lower compared to the categorical models in Gao et al. (2017). The phenomenon could be explained by two possible reasons. Firstly, non-additive effects played important roles in controlling the plant traits (Shen et al., 2014). Dominance and epistasis were additionally considered in the previous gene annotation based categorical models (Gao et al., 2017). The impact of non-additive effects on predictive ability could also be seen when comparing the performance of haplotype allele dosage models with categorical epistasis models in Gao et al. (2017). Secondly, the haplotype allele similarity scores could more or less reflect the identical by decent (IBD) between SNP alleles and thus better in measuring relatedness between pairs of individuals (de Roos et al., 2011). However, the advantages on similarity measuring were not always transferred into the predictive ability (Hickey et al., 2013).

However, integrating gene annotation just slightly improved the predictive ability of several traits and did not show any improvement in the rest in the yellow chicken population (Table 5 and Figure S3). The possible reasons were the frequent recombination in the chicken genome (Fulton et al., 2016) and the underlying trait genetic architecture. Generally speaking, haplotype models were more powerful on reflecting real relatedness between individuals. However, the advantages of haplotype derived relatedness matrices could be expected only when haplotypes were better in tracing the underlying recombination events than SNPs. Previous studies have found extensive diversity and large number of recombination hotpots in the chicken genome (Fulton et al., 2016), which shorten the real haplotype blocks and thus linkage disequilibrium based approaches were more suitable for haplotype blocks constructing. In this study, instead of considering linkage disequilibrium, we implemented a strategy similar to Meuwissen et al. (2014), where maximum number of haplotype alleles was used as threshold when adding SNPs to haplotypes, for haploblock constructing. This approach might not be suitable for the species that extensive diversity and abundant recombination existed in the genome. Therefore, linkage disequilibrium based haploblock construction methods (Cuyabano et al., 2015; Da, 2015) should be suggested for such species. Nevertheless, the main focus of this study was to provide methods of building genic similarity relationship matrices, though the haplotype could be defined through various rules. Even the setting of threshold of the number of haplotype alleles harbored in each haploblock was relatively arbitrary, it was an easy way to build haplotypes and good at controlling the number of variables within each haploblock. Actually, LD information was also reflected indirectly by restricting the maximum of haplotype alleles in certain haploblock, since lower LD among consecutive SNPs would increase the number of haplotype alleles rapidly when adding more SNPs to the haploblocks. Moreover, to our knowledge, the LD based haplotype construction method might have problems on inadequate accurate estimations of LD level in small populations and difficulty in selecting LD threshold for combining consecutive SNPs into haplotype.

In this study, the relatedness matrices used for genetic evaluation were constructed by averaging the relatedness based on individual genes, which meant that weights were assigned equally among genes. The underlying assumption of this approach was that all genes contributed equally to the relatedness matrices and thus to the traits. However, abundant accumulative biological knowledge had shown that gene effects were different among traits. Moreover, previous studies had found that genomic prediction models could be improved when genetic architecture was considered by assigning different weights to SNPs (Zhang et al., 2010; Ober et al., 2012; Gao et al., 2015). Therefore, similar approaches to construct trait specific relatedness matrices by weighting genes differently (Zhang et al., 2010; Ober et al., 2012; Gao et al., 2015) in the paradigm of genic similarity genomic prediction models are worth trying in the future.

Overall, we proposed a new strategy to construct relatedness matrices on the gene level by transferring the genic haplotype similarity scores into individual similarity matrices. New explanatory variables on the gene level were derived from phased SNPs and through which the prediction model was moved one step further from SNPs to biologically functional units. The genic similarity matrices based model showed benefit in respect of predictive ability for many traits in the studied populations. However, predictive ability was not improved in some traits, especially in the yellow chicken population, which indicated that the newly proposed approach still had rooms for improvement to adapt different traits or populations. The uniform weight assigned among genes when constructing the relatedness matrices and the insensitivity to genome recombination rate (the strategy for genic haplotype construction) could be the two major limitations of the new approach. Nevertheless, the idea of constructing relatedness matrices on the biologically functional units potentially improved predictive ability.

## Ethics statement

This study was carried out in accordance with the recommendations of Animal Care Committee of South China Agriculture University (Guangzhou, People's Republic of China). The protocol was approved by the Animal Care Committee of South China Agriculture University. Animals involved in this study were humanely sacrificed as necessary to ameliorate their suffering.

## Author contributions

NG and ZZ conceived this study, performed the model validations, and wrote the manuscript. JT and SH helped in the model validations and manuscript. SY, HZ, and XY helped in the manuscript writing. XZ originally derived the yellow chicken data and helped in the analyses. JL stimulated the idea of the paper and helped in the manuscript.

### Conflict of interest statement

The authors declare that the research was conducted in the absence of any commercial or financial relationships that could be construed as a potential conflict of interest.
