# Supplementary material for: Genomic Prediction of Complex Phenotypes Using Genic Similarity Based Relatedness Matrix
Source: Front Genet. 2018 Aug 31;9:364. doi: 10.3389/fgene.2018.00364 (PMC6127733; doi:10.3389/fgene.2018.00364)

FIGURE S1 | Pearson's correlation between observed and predicted phenotypes for 20 replicates of 5-fold cross-validations in the rice breeding population.

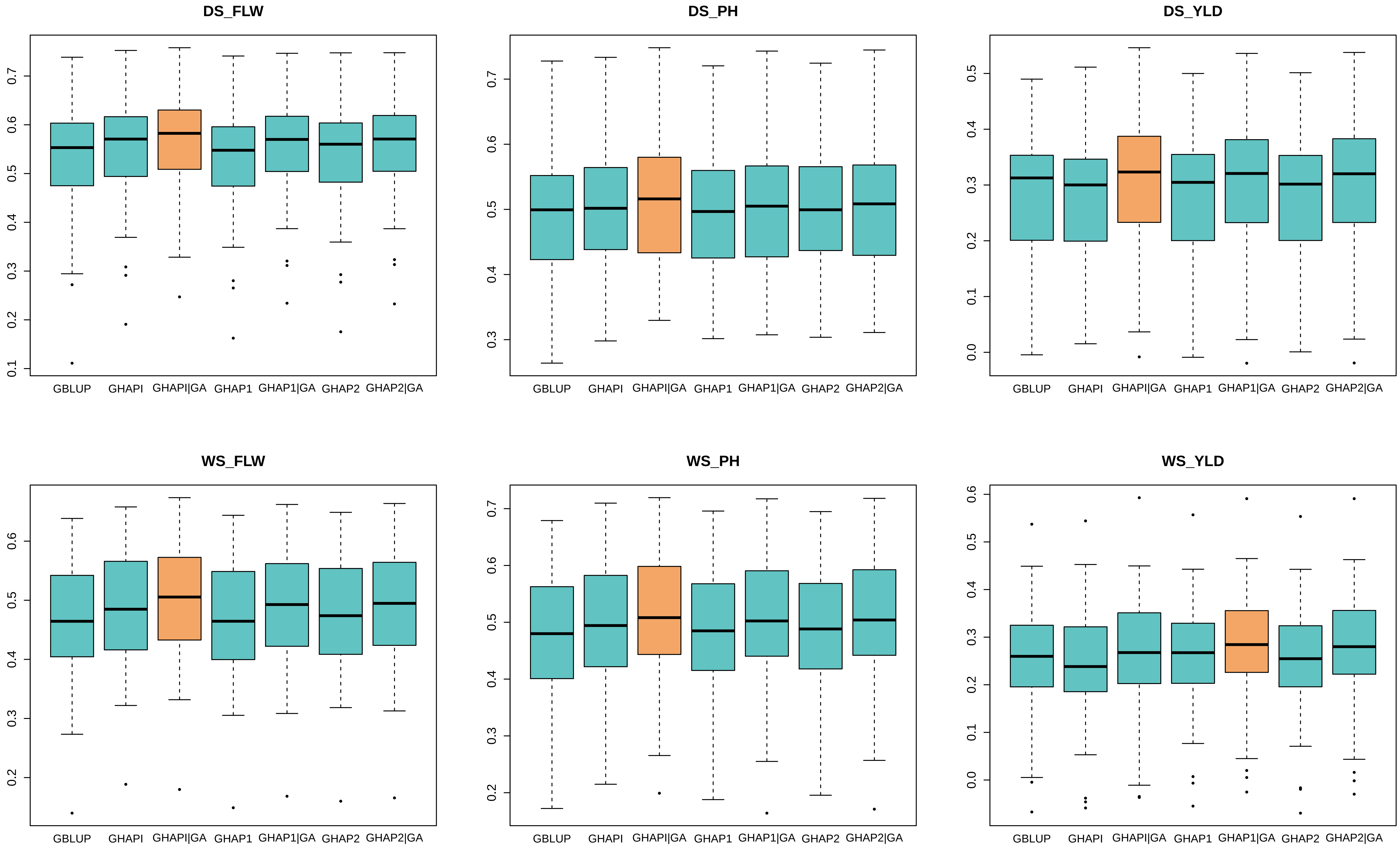

Supplement: Supplementary file 1 [file Data_Sheet_1.pdf]
